# Structural Basis of Inhibition of the Pioneer Transcription Factor NF-Y by Suramin

**DOI:** 10.3390/cells9112370

**Published:** 2020-10-29

**Authors:** Valentina Nardone, Antonio Chaves-Sanjuan, Michela Lapi, Cristina Airoldi, Andrea Saponaro, Sebastiano Pasqualato, Diletta Dolfini, Carlo Camilloni, Andrea Bernardini, Nerina Gnesutta, Roberto Mantovani, Marco Nardini

**Affiliations:** 1 Department of Biosciences, University of Milano, Via Celoria 26, 20133 Milano, Italy; valentina.nardone83@gmail.com (V.N.); antonio.chaves@unimi.it (A.C.-S.); michela.lapi@unimi.it (M.L.); andrea.saponaro@unimi.it (A.S.); diletta.dolfini@unimi.it (D.D.); carlo.camilloni@unimi.it (C.C.); andrea.bernardini@unimi.it (A.B.); nerina.gnesutta@unimi.it (N.G.); mantor@unimi.it (R.M.); 2 Department of Biotechnology and Biosciences, University of Milano-Bicocca, Piazza della Scienza 2, 20126 Milan, Italy; cristina.airoldi@unimib.it; 3 Department of Experimental Oncology, IEO, European Institute of Oncology IRCCS, Via Adamello 16, 20139 Milan, Italy; sebastiano.pasqualato@ieo.it

**Keywords:** transcription factor, histone fold, CCAAT box, NF-Y, suramin, inhibition

## Abstract

NF-Y is a transcription factor (TF) comprising three subunits (NF-YA, NF-YB, NF-YC) that binds with high specificity to the CCAAT sequence, a widespread regulatory element in gene promoters of prosurvival, cell-cycle-promoting, and metabolic genes. Tumor cells undergo “metabolic rewiring” through overexpression of genes involved in such pathways, many of which are under NF-Y control. In addition, NF-YA appears to be overexpressed in many tumor types. Thus, limiting NF-Y activity may represent a desirable anti-cancer strategy, which is an ongoing field of research. With virtual-screening docking simulations on a library of pharmacologically active compounds, we identified suramin as a potential NF-Y inhibitor. We focused on suramin given its high water-solubility that is an important factor for in vitro testing, since NF-Y is sensitive to DMSO. By electrophoretic mobility shift assays (EMSA), isothermal titration calorimetry (ITC), STD NMR, X-ray crystallography, and molecular dynamics (MD) simulations, we showed that suramin binds to the histone fold domains (HFDs) of NF-Y, preventing DNA-binding. Our analyses, provide atomic-level detail on the interaction between suramin and NF-Y and reveal a region of the protein, nearby the suramin-binding site and poorly conserved in other HFD-containing TFs, that may represent a promising starting point for rational design of more specific and potent inhibitors with potential therapeutic applications.

## 1. Introduction

The transcription factor (TF) NF-Y is a nuclear protein that binds the CCAAT sequence in promoters with a very high specificity [1]. The CCAAT box is an important regulatory element, typically located at a conserved distance of −60/−100 bp from the Transcriptional Start Site (TSS) and it is present in 25% of eukaryotic promoters [2]. This occurrence is similar to that of the TATA box, and the CCAAT box is mostly found in TATA-less promoters [2,3]. Genome-wide assays and in vitro experiments have demonstrated that NF-Y is the primary CCAAT-binding protein [4].

NF-Y is a heterotrimer formed by evolutionarily conserved subunits: NF-YA, NF-YB, and NF-YC. NF-YB and NF-YC form a heterodimer via interacting histone fold domains (HFDs), while NF-YA provides DNA sequence-specificity to the trimer. A multitude of genes have been described to be positively or negatively regulated by NF-Y, including prosurvival and cell-cycle-promoting genes, in addition to genes involved in metabolism [5,6,7,8,9,10]. Regarding metabolism, the NF-Y yeast-homologue HAP2/3/4/5 was originally identified as the activator of oxygen-fueled metabolism in the presence of non-fermentable carbon sources, by binding the CCAAT box at Upstream Activating Sequences (UAS) of nuclear genes of the mitochondrial respiratory complexes [11]. In mammals, the NF-Y regulome is far more complex, yet functional dissection of individual promoters suggested the importance of NF-Y for high level expression of metabolic genes: available genomic data and gene expression experiments after inactivation of NF-Y subunits confirmed this [12]. Specifically, following NF-Y removal, expression of anabolic or catabolic genes was found to be reduced or increased, respectively; among the formers, rate-limiting steps in amino acid (Ala, Asp, Glu; Ser, Gly; Gln), lipid (cholesterol and fatty acids) and nucleic acid pathways. Furthermore, as for carbohydrate, carbon metabolism (mostly glycolysis) is almost entirely under NF-Y control. All these metabolic pathways are particularly crucial in cancer cells, where “metabolic reprogramming” is a hallmark of tumor development and progression [13,14,15]. Analysis of expression profiling of large tumor datasets indicate that the NF-Y matrix is enriched in promoters of genes overexpressed in cancer cells [16]. Recently, we, and others, have also reported the overexpression of the NF-YA subunit in different types of tumors and that this correlates with poor disease prognosis [17,18,19,20,21]. It should be noted that while the trimer is found in all growing transformed or immortalized cell lines, cells in specific tissues, particularly post-mitotic ones, lack or contain very low levels of NF-YA. In general, overexpression of oncogenic TFs not only leads to profound and persistent changes in gene expression, but also to the “addiction” of the tumor cell for high TF gene expression level. In such context, a decrease, even if partial, in TF activity, which would normally be marginal in normal cells, could lead to disproportionately higher effects in tumor cells. Based on these premises, NF-Y has been listed among TFs whose targeting could restrict uncontrolled cell growth.

In general, it is known the poor “druggability” of TFs, which lack ligand pockets and act by means of protein–protein and protein–DNA interactions. Yet, attempts to target TFs have been tried with some success [22,23], including employing unbiased screenings without *a priori* knowledge of protein structures [24]. In the absence of the structure of the NF-Y trimer, screening for compounds that inhibit proliferation by targeting the NF-Y/CCAAT complex has mainly focused on minor-groove binding drugs, typically polyamide intercalating derivatives that bind at the preferred NF-Y binding site (i.e., on CCAAT-boxes of the Topoisomerase α promoter) [25,26,27,28,29,30]. More recently, the molecular mechanism of DNA-recognition and binding by NF-Y was revealed at the atomic level via X-ray crystallography [31,32,33]. All three NF-Y subunits were shown to be necessary for DNA binding, covering different roles. The NF-YB and NF-YC subunits dimerize through their HFDs and bind the DNA non-specifically over a long sequence (about 25–30 bp). The NF-YA subunit, once associated to the HFD dimer with its A1 α-helix, recognizes and binds the CCAAT nucleotides via its A2 α-helix and the following Gly-loop, inserting in the minor groove of DNA. These notions represent the basis for more knowledge-based approaches for targeting the subunits and thus NF-Y activity. In fact, recent studies describing the use of peptides that mimic the A1 α-helix of NF-YA, showed their ability to prevent trimer association and therefore CCAAT binding [34].

Here, we present a detailed study of the molecular bases that underlie the possible modulation of DNA binding activity of NF-Y by an already known drug, suramin. Suramin was identified from a large library of pharmacologically active compounds, via in silico docking-simulations carried out on the NF-Y structure. The water solubility of the compound allowed us to test its inhibitory potential without using DMSO, which induces precipitation of NF-Y. Using a combination of biochemical and biophysical approaches, we showed that suramin inhibits the NF-Y/DNA interaction by binding to the HFD of the NF-YB/NF-YC subunits. This demonstrates that NF-Y presents at least one ligandable surface, thus creating the starting point for the rational design of new antiproliferative compounds.

## 2. Materials and Methods

### 2.1. In Silico Search for NF-Y Inhibitors

The virtual Library of Pharmacologically Active Compounds (LOPAC^®^1280) employed for the docking analysis was provided by Sigma-Aldrich and included 1280 commercially available compounds (https://www.sigmaaldrich.com). The AutoDock4 software package [35] was used for a docking screen of the LOPAC^®^1280 library. The Python Molecule Viewer 1.5.6 of the MGL-tools package (https://ccsb.scripps.edu/mgltools/) was used to analyze the data. The atomic coordinates of NF-Y in complex with DNA (PDB ID 4AWL) [31] were used for docking; both the DNA and the NF-YA subunit were removed prior to in silico screening. Hydrogen atoms and Kollman charges were added using the program AutoDock4. The protein model was then used to build a discrete grid within a box (58 × 94 × 68 grid points, with a spacing of 0.375Å) as the explored volume for the compound docking search. The grid was centered on the DNA binding site and, alternatively, on the NF-YA binding site. Fifty independent genetic algorithm runs were performed for each LOPAC library compound (with 150 individuals in the population and 27,000 generations). The docking poses produced were ranked based on the predicted binding free-energy values ΔG (kcal/mol).

### 2.2. Protein Expression and Purification

The recombinant protein constructs for the expression of the minimal functional domains (md) of the NF-Y HFD dimer (YB/YCmd, hereafter NF-Yd), and of the NF-Y trimer (NF-Ymd, hereafter NF-Yt), which constitute the minimal regions for subunit interaction and DNA binding (NF-YB, aa 49–141; NF-YC, aa 27–120; NF-YA, aa 262–332-long subunit numbering-), and of NF-YA C-terminal portion (YA3; aa 239–347), were previously described [31,36]. Proteins were produced in BL21 (DE3) *E. coli* cells, exploiting a subunit coexpression system strategy [37] for NF-Yd and NF-Yt, and purified as previously described [31,36,38]. Briefly, soluble expression of NF-Yt and NF-Yd was achieved upon induction with 0.2 mM IPTG, incubating overnight at 25 °C. Cells were lysed by sonication in Buffer A (10 mM Tris-HCl pH 8, 400 mM NaCl, 2 mM MgCl_2_, and 2 mM imidazole). The cell lysate was loaded on a His-Select Nickel affinity column (Sigma Aldrich, St. Louis, MO, USA), and proteins were purified, exploiting the presence of the 6His-tag at NF-YA C-terminus in NF-Yt, and at NF-YB N-terminus in NF-Yd. The His-tag was removed from the target proteins by incubating pooled, peak fractions (proteins elute in Buffer A + 250 mM imidazole) with thrombin (Thrombin CleanCleave kit, Sigma Aldrich, St. Louis, MO, USA), overnight at 20 °C. Cleaved proteins were further purified on a HiLoad^®^16/60 Superdex^®^75 prep grade size-exclusion column (GE Healthcare, Uppsala, Sweden) pre-equilibrated in Buffer B (10 mM Tris-HCl pH 8, 400 mM NaCl, 2 mM DTT) using an Akta chromatography system (GE Healthcare, Uppsala, Sweden). NF-YA, expressed in *E. coli* BL21 (DE3) with a C-terminal His-tag, was purified by Nickel affinity chromatography, as previously described [36]. Analytical size exclusion chromatography studies of NF-Yd in presence of different concentrations of suramin were performed on a Superdex 75 10/300 GL column (GE Healthcare, Uppsala, Sweden) equilibrated in buffer C (10 mM Tris-HCl pH 8, 150 mM NaCl, 2 mM DTT).

### 2.3. Electrophoretic Mobility Shift Assays (EMSA)

**Figure 1:**
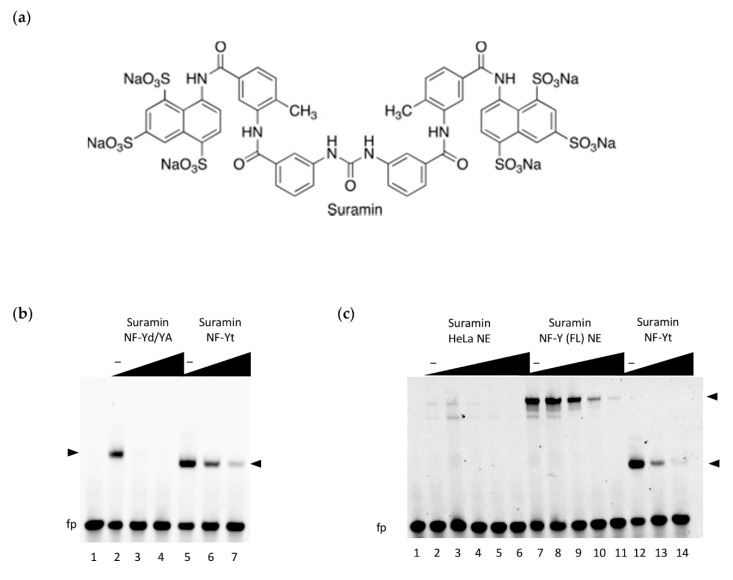
DNA-binding inhibition by suramin; (**a**) chemical structure of suramin; (**b**) DNA binding inhibition of the NF-Y trimer (40 nM) by suramin was assessed by electrophoretic mobility shift assays (EMSA) using a Hsp70 CCAAT box DNA probe (20 nM). Inhibition was tested at increasing doses of suramin (0, 50, 100 µM; lanes 2–4 and 5–7) against the reconstituted trimer, obtained by mixing equimolar ratios of purified NF-YA with NF-Y histone fold domain (HFD) dimer (NF-Yd) (NF-Yd/YA) or on the co-purified trimeric subunit protein (NF-Yt). Lane 1: probe alone DNA binding mix in the absence of NF-Y. NF-Y/DNA complexes are indicated by arrowheads. fp: free probe. Slower migration of NF-Yd/YA/DNA, as compared to NF-Yt/DNA (composed of the minimal DNA-binding domains), reflects the higher molecular weight of the purified NF-YA subunit within the complex; (**c**) EMSA experiments using nuclear extracts (NE) from HeLa cells, obtained from control cells (HeLa NE: lanes 2–6) or from cells overexpressing the full-length NF-Y subunits (including the transactivation domains) (NF-Y(FL) NE: lanes 7–11). NF-Yt recombinant protein was used as a positive control for suramin inhibition of DNA binding (lanes 12–14). In lanes 2–11, the reaction mix includes 2.3 µg of nuclear extract, and an increasing concentration of suramin for NE and NE + NF-Y(FL) (0, 1, 50, 100, and 200 µM), lanes 12-14 reactions include NF-Yt protein (40 nM) and suramin (0, 100, and 200 µM). Lane 1: probe alone DNA binding mix without NF-Y subunits or NE added. The migration of FL and minimal domain NF-Y/DNA complexes are indicated by black arrowheads. “fp”: free probe, “‒”: no suramin.

For EMSA experiments, recombinant proteins were added to a Binding Mix in 16-µL reaction volume, containing a 31-bp Cy5-labelled DNA probe derived from the human HSP70 promoter [31]. The final composition of the binding reaction was: 40 nM protein, 20 nM HSP70 probe, 20 mM Tris·Cl pH 7.5, 50 mM NaCl, 5 mM MgCl_2_, 0.5 mM EDTA, 6.5% glycerol, 2.5 mM DTT, 0.1 mg/mL BSA. Suramin (Sigma-Aldrich, St. Louis, MO, USA—cat no. S2671) was included at indicated concentrations in the binding mix (containing NF-Yd in the case of reconstituted trimer reactions), before the addition of recombinant proteins (NF-YA, or NF-Yt), or nuclear extracts. Reactions were assembled on ice and incubated at 30 °C for 30 min in the dark. An aliquot of each reaction was loaded on a 6% non-denaturing polyacrylamide gel and run in 0.25× TBE at 80 V at 4 °C. EMSAs with HeLa cell nuclear extracts were performed essentially as previously described [36]: to obtain nuclear extracts for NF-Y overexpression and control samples, HeLa cells were grown at 37 °C in DMEM high glucose with L-glutamine and 10% FBS (EuroClone, Pero, MI, Italy) and seeded in 6-cm plates; the next day, cells were co-transfected with 350 ng of each full-length NF-Y subunit expression vector (pSG5-NF-YA, pSG5-NF-YB, pCMV2-flag-NF-YC), or empty control plasmid, to a total of 2.3 µg DNA. After 24 h, cells were harvested for nuclear extract preparation as described in [39]. Nuclear extracts from transfected and control cells were used in EMSA as described in [36]. For EMSAs shown in Figure 1b,c, suramin inhibition of DNA binding was assayed in three independent experiments, using the same preparation of nuclear extracts for Figure 1c.

### 2.4. Isothermal Titration Calorimetry (ITC)

Experiments were performed at 25 °C using a VP-ITC MicroCalorimeter (MicroCal, Malvern Instruments Ltd., Malvern, UK) following the general procedure, as previously described [40]. Briefly, the volume of the sample cell was 1.4 mL; the reference cell contained water. Suramin (250 µM) was titrated using injection volumes of 8 µL into a solution containing the required protein at 20 µM. Both protein and suramin were diluted with the same buffer to obtain a final solution in Buffer C (10 mM Tris-HClpH 8, and 150 mM NaCl). Calorimetric data were analyzed with the software packages NITPIC, SEDPHAT, and GUSSI [41].

### 2.5. Saturation-Transfer Difference (STD) NMR

NMR spectra were acquired on a Bruker AVANCE III 600 MHz NMR spectrometer equipped with a QCI (^1^H, ^13^C, ^15^N/^31^P, and ^2^H lock) cryogenic probe. Samples for STD NMR experiments were prepared as follows: the stock solution of protein (NF-YB/NF-YC or trimer in 10 mM Tris-DCl and 0.4 M NaCl, pH 8) was diluted to 25 µM in the NMR sample and the stock solution of suramin (5 mM) in D_2_O was diluted to 1 mM in the NMR sample. The final concentration of the buffer was brought to 10 mM Tris-DCl and 150 mM NaCl in the NMR sample, changing gradually the ionic force over 30 min and keeping the sample at 4 °C. Samples containing the small molecule (1 mM) in 10 mM Tris-DCl and 0.4 M NaCl, pH 8, were also prepared to record the corresponding 1H-NMR and STD NMR blank experiments. The total sample volumes were 560 µL. The pH of each sample was measured with a microelectrode (Mettler Toledo, Columbus, OH, USA) for 5 mm NMR tubes and adjusted to pH 8 with small amounts (few microliters) of NaOD and/or DCl. All pH values were corrected for the isotope effect. The acquisition temperature was 25 °C. ^1^H NMR spectra were recorded (*zgesgp* pulse sequences in Bruker library) with 64 scans, with a spectral width of 12 ppm, and a relaxation delay of 3 s. 1D STD NMR spectra were recorded (*stddfiffesgp*.3 pulse sequences in Bruker library) with 1024 scans, with a spectral width of 12 ppm, and saturation times of 3 s, 2 s, 1 s, 0.6 s, 0.3 s, with on-resonance frequency = −1.0 ppm and off-resonance frequency = 40 ppm. They were processed with a line broadening of 0.2 Hz and corrected for phase and baseline.

### 2.6. Crystallization, Data Collection, Structure Determination and Refinement

Suramin sodium salt (catalogue No. S2671) was obtained from Sigma–Aldrich (St Louis, MO, USA). After overnight incubation at 4 °C of NF-Yd with a tenfold molar excess of suramin in Buffer B, crystals of the complex NF-Yd/suramin were prepared at 20 °C in 200 mM ammonium citrate tribasic, and 20% (*w*/*v*) polyethylene glycol 3350, using the sitting-drop technique. Crystals were cryoprotected in the same reservoir solution supplemented with 20% (*w*/*v*) glycerol before cooling in liquid nitrogen. X-ray diffraction data were collected at the ESRF synchrotron (ID29 beamline, Grenoble, France). The crystal diffracted at 2.7 Å with the *P*2_1_2_1_2_1_ space group, with two molecules of NF-Yd and one molecule of suramin per asymmetric unit. Raw data were processed with XDS [42] and Scala [43]. The structure was solved by molecular replacement using the software Phaser [44], and the NF-YB/NF-YC dimer as the search model (PDB-code 4CSR). Iterative cycles of model building with Coot [45] and refinement with Refmac5 and Phenix [46,47] were carried out to produce the final model. The stereochemical parameters of the final model were checked with Molprobity [48]. Data processing and refinement statistics are summarized in Table 1. Atomic coordinates and the structure factors have been deposited in the Protein Data Bank (www.rcsb.org) with entry code 7AH8.

### 2.7. Molecular Dynamics (MD) Simulations

NF-Yd was described by the Amber99SB force-field and solvated in ~23,000 TIP3P water molecules [49]. Suramin was parametrized using GAFF2 [50]. Partial charges were derived by a density functional theory B3LYP all-electron calculation on a 6-31G** basis set by RESP. Density functional calculations have been performed using CP2K [51] while MD simulations were performed using GROMACS 2016 [52]. The initial conformation of the system was taken from the symmetrical dimer found in the crystal and solvated in a dodecahedron box of 735 nm^3^. Short-range Coulomb and van der Waals interactions were cut-off at 0.9 nm with long-range Coulomb interactions treated using Particle Mesh Ewald. After energy minimization, the temperature and density of the system were equilibrated keeping the NF-Yd/suramin position fixed for 1 ns. A production 1.2 µs simulation was run at 300K and 1 atm using the Bussi’s thermostat [53] and the Parrinello-Rahman barostat [54].

## 3. Results

### 3.1. Identifying Suramin as a Compound Binding to NF-Y

Virtual screening was initially set up to discover synthetic compounds that interfere with NF-Y activity. Docking simulations were carried out using the NF-YB/NF-YC HFD dimer (NF-Yd: derived from the 4AWL PDB structure) as a receptor. The volume target for binding included the NF-Y trimerization interface, a mostly negatively charged groove of NF-Yd, or the DNA-binding surface, a wide mostly positively charged region (Supplementary Figure S1). These regions were explored using a library of small molecules as described in Materials and Methods. The docking search produced a list of compounds with predicted binding free-energy values (ΔG) up to −12.3 kcal/mol. Among the ten top ranking compounds, we noticed that suramin (ΔG = −12.1 kcal/mol; Figure 1a) was the only compound that is water-soluble, while the other ligands were soluble only in DMSO. Dynamic Light Scattering experiments indicated that DMSO (1–10%) induces high polydispersity (>25%) and non-specific aggregation of NF-Yd (1 mg/mL) (data not shown), thus precluding any reliable biophysical characterization of the NF-Y-binding activity for DMSO-soluble compounds. For this reason, we focused our attention on suramin, which was assayed in its ability to inhibit NF-Y function in EMSA.

### 3.2. Inhibition of NF-Y DNA-Binding by Suramin

To evaluate whether the suramin can inhibit the specific binding of NF-Y to DNA, we firstly performed EMSA experiments with purified NF-Y recombinant proteins, using a fluorescently labeled high-affinity CCAAT box probe derived from the human HSP70 promoter [31]. EMSA binding reactions (Figure 1b) were assembled in the presence of increasing concentrations of suramin, either using the reconstituted NF-Y trimer (NFYd/YA), obtained by combining equimolar ratios of the individually purified NF-YA and the HFD co-expressed dimer (NF-Yd) proteins, or with the co-expressed minimal NF-Y DNA binding domain trimer (NF-Yt). We observed that in both cases, suramin addition substantially decreased the formation of NF-Y-bound DNA complexes, in a dose-dependent manner. Suramin’s action is more evident when the combined NF-YA and NF-Yd proteins are assayed, as compared to the pre-formed NF-Yt, suggesting that the C-terminal region of NF-YA (not involved in trimerization and demonstrated to be flexible in the absence of DNA [32]) might partly interfere with suramin binding before repositioning itself at the CCAAT for DNA binding, thus decreasing the effective concentration of the inhibited protein. Considering that the tested constructs are composed of the minimal DNA-binding homology regions of NF-Y, in order to ascertain whether suramin functional inhibition could also hold true on full-length (FL) native proteins expressed in mammalian cells, we performed further EMSAs in which the recombinant proteins were replaced with nuclear extracts. To obtain a significant (specific) signal of NF-Y-bound DNA complexes, HeLa cells were transfected with NF-Y subunit vectors (NF-Y(FL) NE) to obtain extracts that overexpress the three full-length subunit proteins. As a comparison, the NF-Yt recombinant protein was assayed in parallel, together with control (untransfected cells) nuclear extracts (HeLa NE). The results demonstrate that, in the presence of all nuclear components of the mammalian cell (HeLa) extracts, suramin addition efficiently interferes with DNA binding of the native NF-Y FL protein, similarly to NF-Yt (Figure 1c). Importantly, suramin can modulate the binding of NF-Y to DNA at similar concentration ranges as observed for the recombinant protein.

### 3.3. Interaction between NF-Yd and Suramin

**Figure 2:**
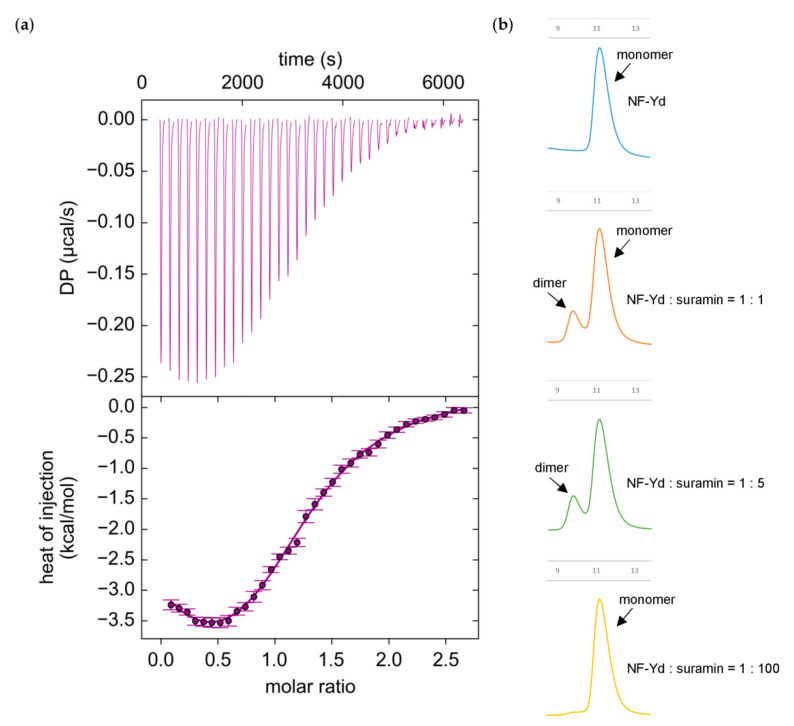
Biophysical analysis of suramin binding to NF-Yd; (**a**) Suramin binding to purified NF-Yd proteins measured by isothermal titration calorimetry (ITC). Upper panel, heat changes (µcal/s) during successive injections of 8 µL of suramin (250 µM) into the chamber containing NF-Yd (20 µM). Lower panel, binding curve obtained from data displayed in the upper panel. The peaks were integrated and normalized per mole of suramin injected. Both panels are plotted against the molar ratio suramin:NF-Yd. The solid line represents a nonlinear least-squares fit to a double dependent sites binding model (see Materials and Methods). The experiment was repeated three times; (**b**) gel-filtration analysis (Superdex 75 10/300 GL column, GE Healthcare, Uppsala, Sweden) of the oligomerization state of NF-Yd induced by different molar ratios of suramin.

In order to quantify the affinity of suramin for NF-Y, we used isothermal titration calorimetry (ITC). Figure 2a reports a representative ITC experiment in which suramin was injected into a sample cell containing NF-Yd.

The thermogram exhibited a biphasic behavior, indicating the occurrence of at least two binding events. Since it is known that suramin can induce the dimerization of its receptors [55,56], in order to determine the appropriate binding model for fitting ITC data we analyzed the oligomerization state of NF-Yd as a function of the suramin relative concentration by gel filtration (GF) (Figure 2b). In the presence of equimolar concentration or low excess of suramin (fivefold more than the protein), the GF chromatograms displayed a second elution peak, compatible with the formation of a NF-Yd protein dimer. This peak was not present at high suramin molar excess (100-fold more than the protein). Therefore, it appears that at low (limiting) concentrations suramin promotes dimerization of NF-Yd, while at high molar ratios suramin saturates all NF-Yd molecules, preventing NF-Yd dimer formation.

Based on these data, we designed a double-dependent binding site model where the binding of suramin to NF-Yd forms a ligand–receptor complex, which in turn generates a binding surface for a second NF-Yd molecule. As suramin concentration increases, the suramin-induced dimerization of NF-Yd is penalized (for details see Material and Methods). The proposed model well fits the ITC data (solid line in Figure 2a) yielding two mean dissociation constants (K_d_) values of 2.9 ± 0.7 µM and of 61.5 ± 9.4 µM for the first (NF-Yd + suramin) and the second (NF-Yd-suramin + NF-Yd) binding event, respectively. Both dissociation constants are in the low micromolar range, with suramin–NF-Yd interaction twenty-fold stronger than the suramin-induced NF-Yd dimerization.

Altogether, ITC and GF analyses indicate that suramin binding to NF-Yd has a dimerization side effect on the transcription factor, which is evident only at low suramin concentrations when NF-Yd can bind to both free suramin or to a NF-Yd–suramin complex. In contrast, when each NF-Yd is saturated by suramin, no dimerization between NF-Yd–suramin complexes can occur. This strongly suggests that one suramin can bind to two NF-Yd molecules simultaneously (see also crystallography section).

### 3.4. STD NMR Binding Experiments

**Figure 3:**
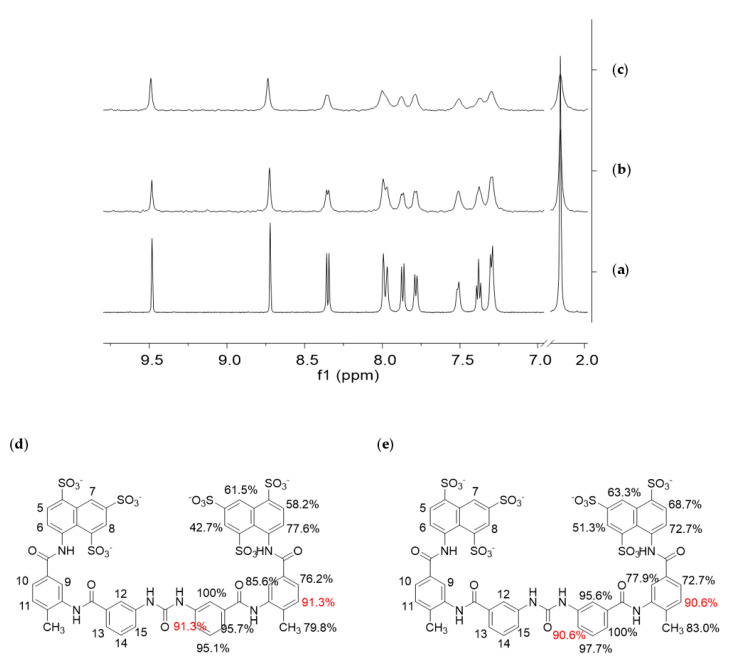
Saturation-Transfer Difference (STD) Nuclear Magnetic Resonance (NMR) experiments on suramin–NF-Y complexes; (**a**) ^1^H-NMR spectrum of suramin 1 mM; (**b**) STD NMR spectrum of a mixture of suramin (1 mM) and NF-Yd (25 µm); (**c**) STD NMR spectrum of a mixture of suramin (1 mM) and NF-Yt (25 µm). All samples were dissolved in 10 mM Tris-DCl, 150 mM NaCl, pH 8, and analyzed at 25 °C and 600 MHz. The ^1^H-NMR spectra were recorded with 64 scans and the STD NMR spectra with 1024 scans and 3-s saturation times; (**d**) binding epitopes of suramin to NF-Yd, and (**e**) trimer obtained for 0.6-s saturation time. The largest relative STD intensity was scaled to 100%. Values are referred to half molecule and are intended duplicated symmetrically. Values in red refer to protons whose overlapping prevented the discrimination of their single contribution.

STD NMR spectroscopy [57,58,59] was employed to characterize the molecular recognition events involving the NF-Yd or NF-Yt and suramin in solution. STD NMR experiments can reveal interactions between a small molecule and a high molecular weight biomolecule, such as a protein or, as in this case, a protein complex, by detecting the transfer of magnetization from the receptor to the ligand that can occur only if both molecular entities bind to each other. Some receptor resonances are selectively saturated and, after binding, the magnetization is transferred from the receptor to the ligand. The detection of ligand NMR signals in the STD spectrum is an unequivocal evidence of its interaction with the receptor. On the contrary, any signal from non-binding compounds is erased in the difference spectrum (STD spectrum). STD NMR spectra (Figure 3b,c) were acquired on samples containing a mixture of the protein (dimer or trimer 25 µM) and suramin (1 mM) dissolved in 10 mM Tris-DCl, pH 8, 150 mM NaCl.

Selective saturation of some aliphatic protons of the protein was achieved by irradiating at −1.00 ppm (on-resonance frequency), a spectral region where no resonances of the putative ligand are present. Figure 3a shows the ^1^H NMR spectrum of the ligand, used as a reference for its characterization, and Figure 3b,c report the STD NMR spectra recorded on the different protein/ligand mixture. The presence of suramin resonances in the STD spectra demonstrates the interaction of the compound with both the dimer and the trimer.

From a qualitative point of view, the stronger intensity of a ligand signal in the STD NMR spectrum indicates shorter inter-proton distances between that ligand proton and the receptor surface in the bound state, allowing the identification of the portion of a ligand most involved in the interaction with a receptor (epitope mapping). Here, STD experiments were acquired with five different saturation times (0.3, 0.6, 1.0, 2.0, and 3.0 s) (data not shown), to obtain the relative STD intensity for each proton of the ligand that gives STD signal. Thus, from these data, the ligand-binding epitopes were characterized, showing to be essentially similar/superimposable for the NF-Yd and NF-Yt (Figure 3b).

### 3.5. NF-Yd–Suramin Complex

To shed light on the molecular mechanisms of NF-Y inhibition described above, we undertook crystallographic analyses of the TF in complex with suramin. The NF-Yd–suramin co-crystals belonged to space group *P*2_1_2_1_2_1_ and diffracted to 2.7 Å resolution. The refinement converged to an R_factor_ value of 22.2% (R_free_ = 27.4%), with a final model composed of two NF-Yd protomers (identified with A/B, and C/D chains for NF-YB/NF-YC, respectively), one suramin molecule, one glycerol, one citrate, and 47 water molecules in the asymmetric unit. After initial refinement cycles of the protein structure, residual electron density was visible for the full molecule of the bound suramin that was modeled accordingly. The refinement statistics and other information are provided in Table 1.

**Figure 4:**
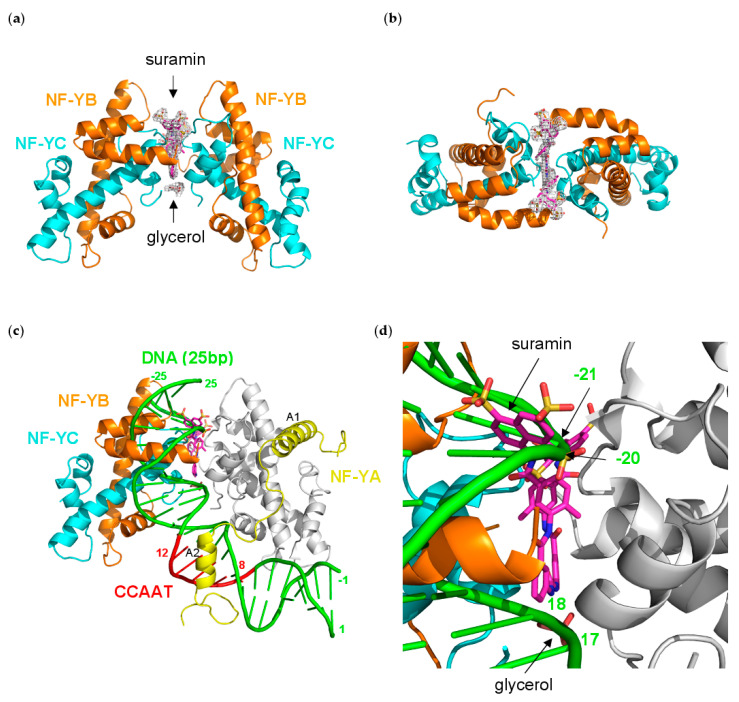
Structure of the (NF-Yd)_2_–suramin complex; (**a**) ribbon diagram showing the bound suramin (magenta sticks) at the dimerization interface of two NF-Yd molecules (NF-YB in orange, and NF-YC in cyan), together with a glycerol molecule (from the cryoprotectant solution). A representative electron density map (grey net), contoured at 1.0 σ, is shown around the bound molecules; (**b**) top view of (NF-Yd)_2_–suramin complex; (**c**) Structural superposition of one NF-Yd molecule of the (NF-Yd)_2_–suramin complex with the DNA/NF-Y complex (NF-YB/NF-YC in grey, NF-YA in yellow, DNA in green) (PDB-code 4AWL). The CCAAT box is shown in red. The NF-YA A1 and A2 α-helices are labeled, and the DNA numbering is indicated. About 10 bp of the bound DNA superimpose with the second NF-Yd molecule of the (NF-Yd)_2_–suramin complex; (**d**) close-up of panel (**c**) showing that two sulfonic acid groups of suramin and the glycerol molecule approximately match the positions of two phosphate groups in both DNA strands of the DNA/NF-Y complex (−20, −21, and 17, 18, respectively).

The suramin molecule adopts an elongated conformation in the (NF-Yd)_2_–suramin complex, and binds at the interface between two NF-Yd protomers, in an almost symmetric manner (Figure 4a,b).

**Figure 5:**
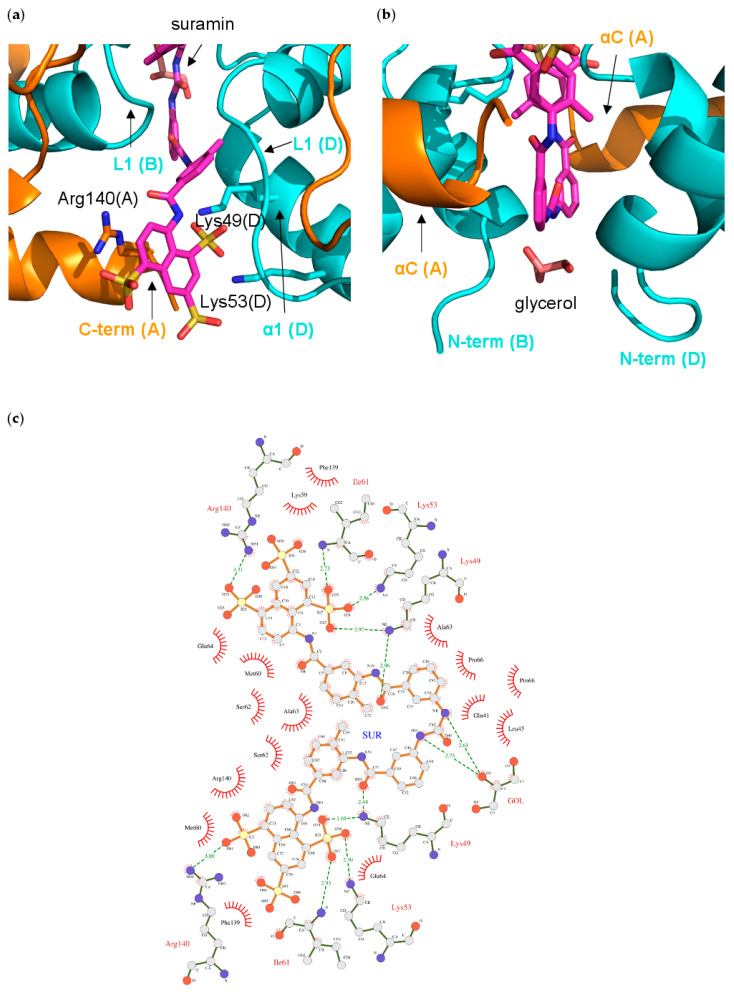
Interactions of suramin in the NF-Yd structure; (**a**) close-up of the binding pocket of half-suramin at the NF-Yd dimerization interface. Secondary structure elements and protein chains are indicated. Color coding as in Figure 4; (**b**) glycerol-binding pocket relative to the suramin binding site. The glycerol molecule is shown in pink sticks; (**c**) schematic representation with LIGPLOT [60]. Polar contacts are depicted as broken lines and hydrophobic contacts are indicated by arcs with radiating spokes.

**Figure 6:**
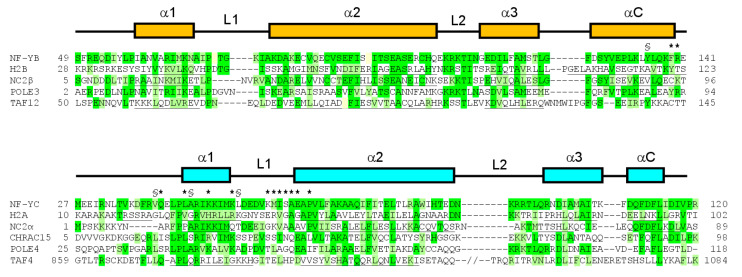
Sequence alignment of NF-YB and NF-YC with HFD proteins; the NF-YB sequence is aligned with human proteins H2B (sp|P06899|; PDB-code 4AFA), NC2β (sp|Q01658|; PDB-code 1JFI), POLE3 (sp|Q9NRF9|), and TAF12 (sp|Q16514|; PDB-code 1H3O). The NF-YC sequence is aligned with human proteins H2A (sp|P04908|; PDB-code 4AFA), NC2α (sp|Q14919|; PDB-code 1JFI), CHRAC15 (sp|Q9NRG0|), POLE4 (sp|Q9NR33|), and TAF4 (sp|O00268|; PDB-code 1H3O). The secondary structure arrangement of the HFD of NF-YB and NF-YC is shown above the sequences. When available, secondary structure information for the other aligned proteins was included (helical residues underlined). In TAF4, the predicted α3 region of the sequence is also aligned, separated by two slashes indicating the ~100 aa loop. Identical residues are highlighted in green, similar residues in light green. NF-Yd residues involved in suramin and glycerol binding are indicated by * and §, respectively.

Half of the ligand molecule establishes interactions in a cleft lined by residues from the NF-YC B and D chains, and from the NF-YB A chain. In detail, the half suramin-binding site is lined by the NF-YC N-terminus (Gln(D)41), helix α2 (Glu(D)64, and Pro(B)66), helix α1 (Leu(B)45, Lys(B)49, and Lys(B)53), and loop L1 (Lys(B) 59, Met(B)60, Ile(B)61, Ser(B)62, and Ala(D)63), and by the NF-YB C-terminal residues (Phe(C)139, and Arg(C)140) (Figure 5a,c and Figure 6).

In particular, the charges of two out of the three sulfonates are compensated by Arg(C)140, and Lys(B)49/Lys(B)53, while the third –SO^3−^ points toward the solvent. Lys(B)49 also interacts with the carbonyl oxygen of the carbonylimino group linking the naphthalene ring and the benzene ring of suramin (Figure 5c). The other half of the suramin molecule binds to the corresponding protein regions of the A, B, and D chains, respectively. Overall, the suramin molecule fits well at the dimeric interface of the two NF-Yd molecules. A further suramin interaction is provided by a glycerol molecule from the cryoprotectant solution, which is symmetrically hydrogen-bonded to both the NH groups of the functional urea moiety (Figure 4a and Figure 5b,c).

Structural comparisons made between the (NF-Yd)_2_–suramin complex and the NF-Yd structure (PDB-code 4CSR) show that, besides promoting the dimeric assembly of the protein, binding of suramin is not associated with any significant tertiary structure modifications (rmsd 0.75 Å). However, local residue adaptations to host the ligand are evident in the C-terminal region of NF-YB (residues 135–141), and in the L1-loop of NF-YC (residues 59–64). In particular, the NF-YB Phe139 side chain rotates by about 90° to accommodate the two benzene rings attached to the urea moiety of the ligand (Supplementary Figure S2).

Structural superimposition with the NF-Y/DNA complex (PDB-code 4AWL) provides hints on the mechanism of DNA-binding inhibition induced by suramin binding (Figure 4c). In the (NF-Yd)_2_–suramin complex, the ligand is located close to the DNA binding region of NF-Y, contacting NF-YC L1 and α1 and NF-YB αC (Figure 5a,b), which are responsible for more than 40% of the contacts between the HFD NF-Yd and the phosphate backbone of the NF-Y-bound DNA [31]. Furthermore, two sulfonic acid groups that decorate the suramin naphthalene moiety, in particular the one salt-bridged to the NZ atoms of NF-YC Lys49 and Lys53, match well with the position of two DNA phosphate groups, corresponding to position −21 and −20 in the complementary strand of the CCAAT box (Figure 4d) in the DNA/NF-Y complex [31]. Additionally, the suramin-induced homodimerization of the HFD NF-Yd sterically precludes the binding of about 10 bp present in the NF-Y/DNA complex (16−25 in 4AWL) (Figure 4c).

On the contrary, suramin binding seems not to interfere with NF-Y trimerization, since the interaction surface between NF-YA and the NF-Yd (NF-YB α2, NF-YC L2 and αC) is distant from the suramin-binding site (Figure 4c). These results are in line with our STD NMR data that indicate that suramin binds in a similar manner to both NF-Yd and NF-Yt.

### 3.6. Molecular Dynamics Simulation

**Figure 7:**
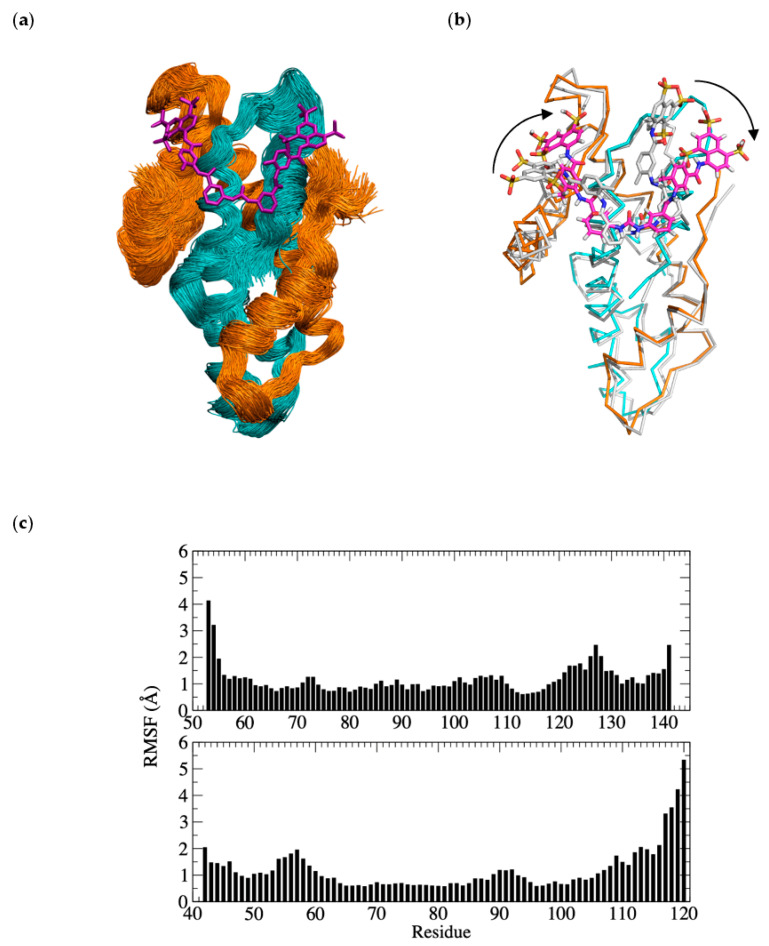
Molecular dynamics (MD) simulations of the NF-Yd–suramin complex; (**a**) superimposed frames (one per ns) for the last µs of simulation (orange NF-YB, cyan NF-YC, and magenta suramin); (**b**) structural superposition of the middle structure of the most populated cluster of the MD simulations (color code as in panel (**a**) with the crystal structure of the complex (grey)). The readjustment of the two naphthalene rings to optimize the interactions with the single NF-Yd molecule are highlighted by arrows; (**c**) root mean square fluctuations (RMSF) per NF-YB and NF-YC residues (upper and lower panels, respectively).

Given that the binding of suramin resulted in the formation of a symmetrical dimer of NF-Yd in the crystal, we probed whether the interaction between suramin and one NF-Yd molecule is sufficient to provide a stable complex or if a sandwich between two NF-Yd protomers is compulsory for suramin binding. To this aim, we performed 1.2 µs MD simulations at 300K. The MD results show that the interaction of suramin with a single copy of the NF-Yd is stable for the full duration of the simulations (Figure 7a,c).

The overall binding mode is preserved with the central urea functional moiety of suramin well-accommodated in the complementary surface groove formed by NF-YC α1, and α2. Diversely, some variations are found for the position of the two naphthalene rings that tend to readjust their position to optimize the interactions with the single NF-Yd molecule (Figure 7b). In the absence of the second (heterodimeric) NF-Yd protomer, the NF-YC L1 reorients its position towards the NF-YC α1 and creates a pocket (lined by Lys49, Lys53, Leu54, and Glu56) that hosts the naphthalene ring of the orphan half suramin molecule. The other naphthalene ring moves towards the NF-YC α2 and NF-YB L2 and binds in a surface cleft lined by NF-YC Ser62, Ala63, Glu64, and NF-YB Arg108, Thr110, Asn112, Glu114, and Phe139 and Arg140 at the NF-YB C-terminus. In the MD simulation, two of the suramin sulfate groups mimic the DNA phosphate backbone as found in the DNA/NF-Y trimer complex.

## 4. Discussion

In this study, we demonstrated that suramin, a polysulfonated naphthylamine derivative of urea, inhibits the DNA interaction of NF-Y with the CCAAT box by binding the HFD subunits, either as isolated heterodimers or in the complete trimer.

The first issue concerns whether NF-Y is a reasonable target for pharmacological anti-cancer intervention. The NF-Y subunits are evolutionarily conserved, from yeast to plants to mammals, as is the NF-Y binding site, the CCAAT box, which is relatively widespread in human promoters (about 25%). The three NF-Y subunits are expressed widely in many (but not all) tissues. This implies that to target cancer cells, NF-Y drugs may be too “unspecific”, or too toxic, as they are likely to also heavily affect normal cells. However, a specific role of NF-Y in cancer progression recently emerged. First, the CCAAT box is selectively present in cell-cycle and metabolic genes. Furthermore, essentially all G2/M genes, whose expression is typically altered in cancer, and genes relating to certain metabolic pathways (lipids, glycolysis, nucleotides, SOCG, and glutamine) show altered expression. This is essentially the reason why bioinformatics analyses of promoters of genes overexpressed in cancers often rank CCAAT at the top of the list; in down-regulated genes, instead, CCAAT has never been found. Second, it is becoming clear that NF-YA is robustly and widely overexpressed in epithelial cancers and this is correlated to a worse tumor prognosis [18,19,20,21]. Third, with the possible exception of the hematopoietic system, mice models with conditional KO of NF-YA in different cell types (adipocytes, neurons, hepatocytes) showed chronic, but not abrupt, acute effects [61]. Fourth, metabolic rewiring of cancer cells is associated with, and possibly caused by, overexpression of genes at crucial crossroads in the nucleic acid, amino acid, glucose and lipid metabolic pathways, the vast majority of which are under NF-Y control, based on subunits inactivation and genomic location analysis [12]. Explicative of this is our recent dissection of the glutamine biosynthetic pathway: tumor cells that are sensitive to glutamine starvation become more resistant upon NF-YA stable overexpression entailing up-regulation of CCAAT-dependent genes [62]. In summary, in agreement with other active studies in this field, NF-Y may represent an interesting target for anti-cancer therapy. To date, two directions have been pursued: inhibition of selected sites by DNA-binding drugs [25,26,27,28,29,30] and, following the 3D structure determination [31], interference of trimerization by modified peptides [34]. A third approach, also possible due to structural knowledge, is described here, based on the NF-Y–suramin interaction.

Pharmacologically, suramin has been used for the treatment of early stages of human sleeping sickness and onchocerciasis, an infectious cause of blindness [63]. It is also an inhibitor of reverse transcriptase of retroviruses [64], and, because of the antiproliferative effects, it is currently considered for use as an anticancer agent and chemosensitizer in cancer therapy [65,66]. The antiproliferative mechanism(s) of suramin is far from understood, as its activity has been linked to inhibition of various, and disparate pathways. (i) Suramin can dissociate receptor-bound growth factors, consequently resulting in loss of signal transduction [67]. (ii) Suramin and its analogues have been shown to inhibit Hpa in many human cancer cell lines [68,69,70,71]. (iii) Recently, it has been shown to bind HMGA2 and to potently inhibit DNA interactions, providing new insights into its anti-cancer and anti-metastasis functions, since the expression levels of HMGA proteins are associated with metastasis and poor prognosis for many cancer types [72].

In the case of NF-Y, the binding constants of suramin are modest (K_d_ in the low µM range) but the structural characterization of binding provides room for a rational interpretation of the process and for its improvement. The crystal structure of the complex reveals that suramin binds to the surface of the HFDs, thus promoting homodimerization through the formation of a (NF-YB/NF-YC)_2_–suramin quaternary structure (Figure 4a,b). The presence of this new (NF-Yd)_2_ oligomerization state for HFDs is detectable in solution by gel filtration and is in line with the ITC results, indicating two distinct binding events (Figure 2). Based on our structural evidence, these two events may be attributed to the binding of suramin to the surface of one HFD and to the binding of the second to HFD–suramin, to produce the symmetric “sandwiched” (NF-Yd)_2_–suramin homodimer. This interpretation is in agreement with the MD simulations, indicating that the complex between suramin and a single HFD is stable, in addition to gel filtration experiments, showing that when the protein sample is saturated by suramin forming the NF-Yd–suramin complex, HFDs homodimerization was not further sustained, because the (NF-Yd-suramin)_2_ complex is not feasible. The quaternary structure assembly observed for NF-YB/NF-YC in the presence of suramin is not uncommon, and has already been reported in the literature for ecarpholin S, a Ser49-PLA2 from *E. carinatus* venom [55], for MjTX-II, a myotoxic Lys49-PLA2 from *B. moojeni* [56], and for human NAD^+^-Dependent Deacetylase SIRT5 [73]. This suramin-induced oligomerization behavior is related to its symmetrical chemical structure (Figure 1a), that favors symmetrical protein–protein interactions when bound to the target protein surface.

NF-Y represents the first example of an HFD-containing TF that interacts with suramin. The suramin-binding site is located at the HFD surface involved in DNA interactions within the NF-Y/DNA complex (Figure 4c) [31]. On the contrary, the NF-YB/NF-YC region involved in trimerization with NF-YA is located far from the suramin-binding site (Figure 4c) and, accordingly, our STD NMR data show that suramin can bind to the NF-YB/NF-YC heterodimer and to the trimer in a similar way: the suramin binding epitopes are coherent with the binding shown in the crystallographic complex (Figure 3d,e). Within the HFD heterodimer, NF-YC is the subunit that provides the majority of the suramin-interacting residues: located at the first turn of helix α1 and in the L1 region, they are mostly apolar and generate the homodimeric cleft where suramin symmetrically fits (Figure 5a,c and Figure 6). Other residues, i.e., Lys49 and Lys53, provide electrostatic interactions to the suramin sulfonic acid group, matching the position of DNA phosphate groups of the CCAAT complementary strand in the NF-Y/DNA complex (−21 and −20 in 4AWL) (Figure 4d). Additionally, the C-terminus of the NF-YB subunit, in particular Arg140, provides an additional electrostatic interaction to stabilize a second suramin sulfonic acid group (Figure 5a,c). Interestingly, a glycerol molecule is bound to the NH groups of the urea functional moiety of suramin (Figure 5c), with two hydroxyl groups almost matching the position of two phosphate groups of the CCAAT strand in the NF-Y/DNA complex (17 and 18 in 4AWL) (Figure 4d). The pocket hosting the glycerol is solvent-exposed and lined by residues belonging to the N-terminal region of NF-YC (Val40, Gln41, Leu45, and Ala46), but also to the C-terminal region of NF-YB (Tyr135, and Phe139) (Figure 5b and Figure 6). Furthermore, the suramin-induced homodimerization of the HFD heterodimers sterically precludes binding of about 10 bp present in the NF-Y/DNA complex (Figure 4c). Our tests on nuclear extracts obtained from HeLa cells overexpressing full-length NF-Y provide the first validation that the presence of other protein domains (including the transactivation domains) of the native protein, or other components present in HeLa cells nuclear fractions, do not substantially affect the suramin inhibitory activity, as it can efficiently modulate NF-Y binding to DNA in the same molar concentrations as observed with the recombinant protein (Figure 1c).

Due to its large, flexible, and multifunctional nature, suramin tends to be a nonselective drug. In the case of NF-Y, an issue may be its interaction with other HFD-containing proteins [74]. Sequence alignment of NF-YB and NF-YC with other human HFD-containing proteins indicates that the NF-Y suramin-binding site is lined by residues that are partly conserved (Figure 6). The conservation is located to NF-YC α1 and α2 and NF-YB L2, while differences are present at the NF-YC N-terminus and L1 and at the NF-YB C-terminus; based on our crystallographic data, these latter regions line the glycerol-binding cleft. Such areas with low sequence homology with other HFD proteins could be used to drive chemical modifications on suramin to generate either symmetrical or asymmetrical compounds that can improve ligand-binding affinity, and/or selectivity.

In conclusion, many proof-of-concept experiments have suggested various TFs as promising therapeutic targets. As cancer cells can become addicted to the activity of specific oncogenic TFs for survival, then their inhibition can lead to the selective killing of cancer cells compared with normal cells. Targeting TFs has traditionally been challenging due to disordered structures and the necessity to modulate large protein–protein or protein–DNA interfaces. Screening large compound libraries may generally select hit compounds that inhibit the transcriptional activity or may be designed to home in on a specific mechanism of action. This approach has further hurdles due to limited cell internalization of the hit, difficult or even impossible improvement depending on its chemical nature, and, most importantly, off-target activity when the hit is tested in cells. Certainly, an important requirement to increase the probability of success is a more precise knowledge of the structure and mechanism of action of a TF interacting with its cognate DNA sequence (or protein partners). In this context, our data provide a clear picture that dissects the molecular mechanism of NF-Y inhibition by suramin, thus setting the rationale grounds for the development of new potential NF-Y inhibitory compound(s), with improved properties for in vivo testing.

## Figures and Tables

**Table 1:** Data collection and refinement statistics.

**Data Collection**	
Space group	*P*2_1_2_1_2_1_
Cell dimensions	
*a*, *b*, *c* (Å)	45.697, 61.213, 123.533
α, β, γ (°)	90.00, 90.00, 90.00
Resolution (Å)	45.7–2.7 (2.83–2.70) *
Unique reflections	10061 (1299)
*R*merge (%)	0.11 (0.75)
*I*/σ(*I)*	16.2 (3.5)
Multiplicity	12.1 (12.8)
Completeness (%)	100 (100)
**Refinement**	
R_work_/R_free_ (%)	22.2/27.4
No. residues/molecules	
NF-YB	88 (A chain); 89 (C chain)
NF-YC	79 (B chain); 80(D chain)
Suramin	1
Glycerol	1
Citrate	1
Water	47
B-factors (Å^2^)	55.1
R.m.s. deviations	
Bond lengths (Å)	0.009
Bond angles (°)	1.41
Ramachandran statistics	
allowed region (%)	98.1
favorably allowed region (%)	1.9
outliers	0

* Highest resolution shell is shown in parenthesis.

## Supplementary Materials

The following figures are available online at https://www.mdpi.com/2073-4409/9/11/2370/s1, Figure S1: Electrostatic surface of NF-Yd, Figure S2: Structural changes induced by suramin binding.
