# Supplementary material for: Structural Basis of Inhibition of the Pioneer Transcription Factor NF-Y by Suramin
Source: Cells. 2020 Oct 29;9(11):2370. doi: 10.3390/cells9112370 (PMC7692634; doi:10.3390/cells9112370)
Supplement: Supplementary file 1 [file cells-09-02370-s001.pdf]

cells

MDPI

Supplementary Figures

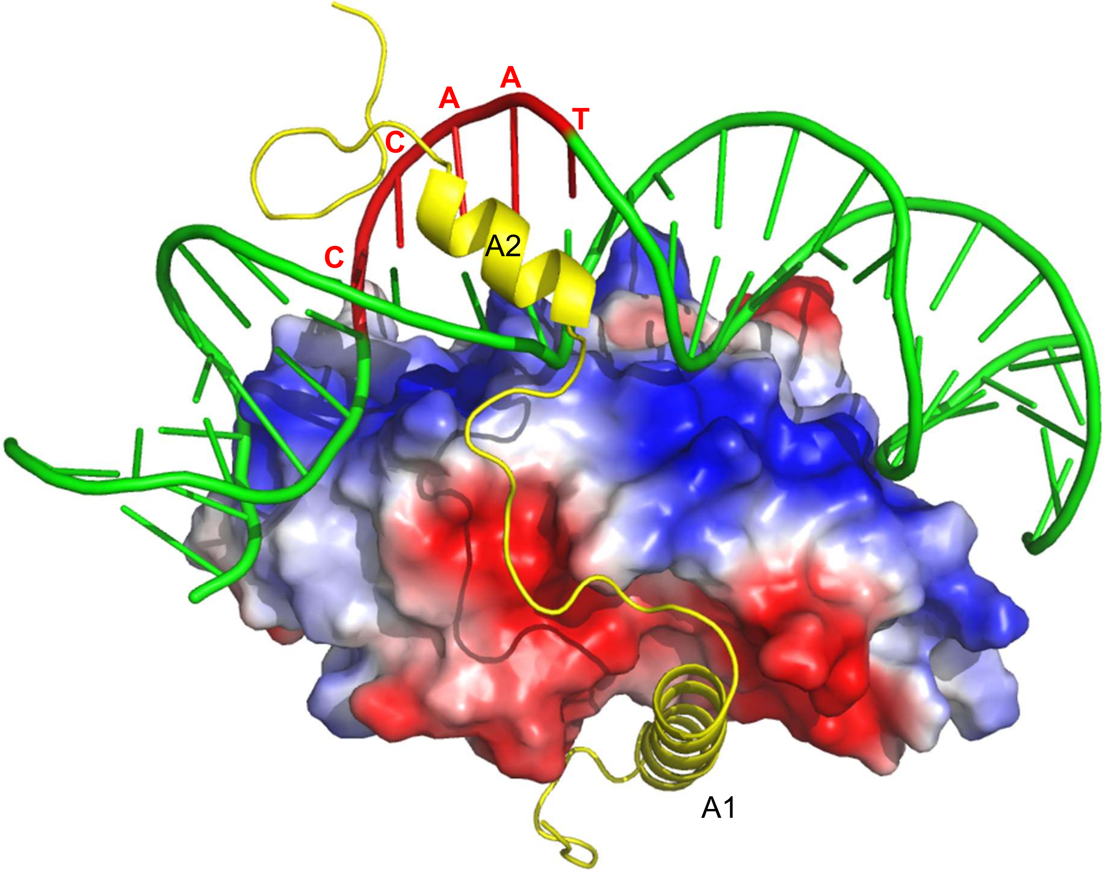

Figure S1. Electrostatic surface of NF-Yd; Blue and red colors indicate positively and negatively charged regions, respectively. NF-YA (yellow) and DNA (green) are represented in ribbon and stick models. NF-YA secondary structure elements (the A1 and A2 α helices) are labeled, and the CCAAT nucleotides highlighted in red.

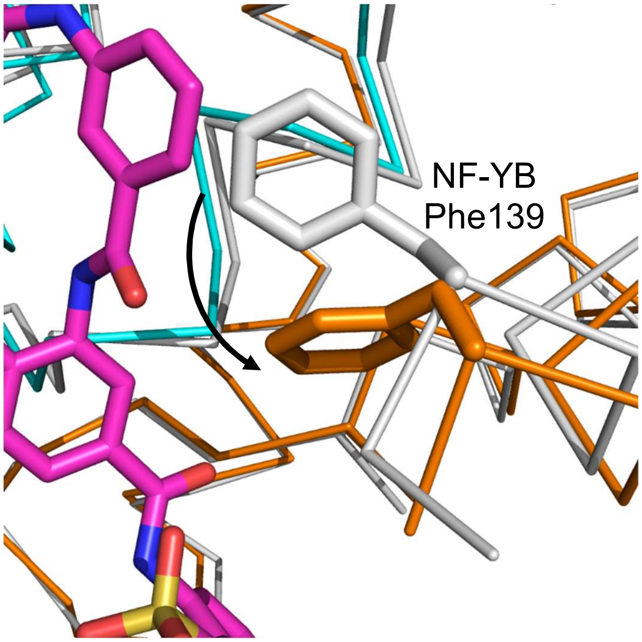

Figure S2. Structural changes induced by suramin binding; rotation (shown with an arrow) of the NF-YB Phe139 side-chain (orange) upon suramin (magenta sticks) binding, relative to its position in the ligand-free NF-Yd (PDB-code 4CSR).
